# Sex-Associated Differences in Outcomes in Acute Myeloid Leukemia Patients Following Intense Induction Treatment: A Real-World Single Center Analysis

**DOI:** 10.3390/jcm14186457

**Published:** 2025-09-13

**Authors:** Xenia Darphin, Janina Blattner, Michèle Hoffmann, Katrin Gobat, Marie-Noëlle Kronig, Thomas Pabst, Berna C. Özdemir

**Affiliations:** 1 Department of Hematology, Spital Limmattal, 8952 Schlieren, Switzerland; 2 Department of Medical Oncology, Inselspital—Bern University Hospital, University of Bern, 3010 Bern, Switzerland; 3 Swiss Group for Clinical Cancer Research (SAKK), 3008 Bern, Switzerland

**Keywords:** sex, acute leukemia, AML, toxicity, chemotherapy, survival

## Abstract

**Background:** Acute myeloid leukemia (AML) is a challenging disease due to its aggressive nature and interindividual variability in treatment response. While sex differences in AML incidence and outcomes have been reported, the impact of sex on treatment-related toxicity remains underexplored. **Methods:** We retrospectively analyzed patients who received intensive chemotherapy for de novo or secondary AML at a single academic hospital between 1 January 2017 and 30 November 2021. A total of 65 patients (62% males) were included, with similar risk categories across sexes. **Results**: Although adverse event rates were similar, females had significantly higher rates of neutropenic colitis (100% vs. 80%, *p* = 0.019) after the first induction cycle. Hematological recovery after the second cycle was faster in males for neutrophils (21 vs. 28 days, *p* = 0.025) and thrombocytes (27 vs. 37 days, *p* = 0.02). Hospitalization duration was also significantly longer for females (26 days vs. 24 days, *p* = 0.039). The median overall survival did not differ by sex, but was significantly longer for those receiving two cycles vs. one cycle (0.5 years vs. NR) and for those <60 years old (6.1 vs. 1.0 years). **Conclusions:** These findings suggest sex-related differences in treatment toxicity and hospital stay length. Larger studies are needed to better understand the impact of sex on AML outcomes.

## 1. Introduction

Acute myeloid leukemia (AML) is an aggressive hematological malignancy with significant clinical and molecular heterogeneity, leading to differences in disease progression, responses to treatment, and a risk of relapse. The median age at diagnosis is about 70 years. It can arise de novo or manifest as a transformation of myelodysplastic neoplasms (MDS) [1,2].

The outcome of AML patients has not significantly improved over the last three decades despite the considerable progress made in targeted therapies [3].

In the past, AML was divided into subtypes according to the French–American–British (FAB) classification, which was based on myeloid differentiation and morphologic findings [4]. Currently, the World Health Organization (WHO) 2022 and International Consensus Classification (ICC) focus on genetic abnormalities, and require a bone marrow blast cut-off of 10–20% [1,5]. Risk groups are divided into favorable, intermediate, and adverse depending on genetic factors, and while patient-related factors are discussed, they are not incorporated in the risk groups according to the European Leukemia Net (ELN) 2017 and 2022 recommendations [6,7].

Treatment for younger, fit AML patients typically consists of an intense chemotherapy induction, which is made up of a backbone of an anthracycline combined with cytarabine, known as “3 + 7”, with the goal of achieving complete remission. This induction regimen has shown cure rates of 30 to 40% in patients <60 years [8]. Depending on the molecular subgroup, newer targeted drugs, such as FLT3 inhibitors or antibody-drug conjugates, can be added to this standard treatment. The induction therapy is generally followed by a consolidation with autologous or allogenic hematopoietic stem cell transplant (ASCT/alloHSCT) or intermediate-dose cytarabine consolidation cycles, depending on the risk group and response to previous treatment [9,10].

Globally, AML is more common in the male population, with an incidence of around 4.5 per 100,000 males compared to 3.0 per 100,000 females [11]. Across different ancestries and age groups, with the exception of those >75 years of age, male sex represents a poor prognostic factor [12]. These sex differences are partially explained by the sex-specific mutational patterns that are present at the stage of clonal hematopoiesis [13,14] and persist throughout disease progression, resulting in distinct disease biologies. Indeed, several analyses found that the distribution of molecular genetic aberrations in AML was sex dependent. While male patients have a higher prevalence of myelodysplasia-related cytogenetic abnormalities, such as *ASXL1*, *RUNX1*, *SRSF2*, and *U2AF1*, females more often have *NPM1*, *FLT3-ITD*, and *CEBPA* mutations [15,16,17]. As a result, more female patients are categorized in the ELN favorable-risk group compared to males [17], showing that these genetic differences are of prognostic significance. In the Chinese cohort reported by Li et al., male patients with favorable-risk AML had lower complete remission rates after the first induction therapy and a higher two-year cumulative incidence of relapse compared to females. In the adverse-risk group, males also had inferior two-year relapse-free and overall survival (OS) rates [16]. Beyond the genetic and survival differences, sex disparities have been observed in the quality of life among AML patients. A study assessing quality of life during treatment found that female patients reported lower physical and functional well-being compared to males, indicating the need for sex-specific support strategies during AML treatment [18]. 

Although emerging data suggest that female patients experience a greater impact of treatment on quality of life and symptoms [19], it is currently not known to what extent patient sex influences treatment toxicity and response. To address this knowledge gap, we evaluated the impact of sex on AML treatment patterns, response, toxicity, and survival in this retrospective study from a single center in Bern, Switzerland.

## 2. Materials and Methods

### 2.1. Study Design and Patient Selection

This retrospective analysis included patients who had received intense chemotherapy induction for de novo or secondary AML at the Inselspital, University Hospital Bern, Switzerland, between 1 January 2017 and 30 November 2021. The follow-up data cut-off was 14 December 2022, and the median follow-up for all patients was 3.3 years.

All patient data were obtained via a search function of electronic medical records containing the induction medication regimes and were de-identified. The cohort of patients included patients ≥ 18 years who were diagnosed with AML. Patients with secondary AML, defined as AML that is secondary to myelodysplastic neoplasms (MDS) or antecedent myeloid hematological disease, as well as treatment-related AML, were eligible for inclusion. Patients with acute promyelocytic leukemia (APL) as well as unfit patients receiving palliative treatment with hypomethylating agents were excluded.

Data from patients who had signed the written general consent of our institution were included. As required by the Ethics Committee of the Canton Bern, for patients alive without a documented general consent, a letter explaining the study and a general consent form were sent to their address. Patients who did not send a refusal of the consent after 30 days were included in the analyses. Patients with a documented refusal were excluded. The study protocol was approved by the Ethics Committee of the Canton Bern (2021-02353).

AML classification at diagnosis was assessed according to the French–American–British Classification (FAB). Based on molecular genetics and karyotyping of the AML cells, patients were classified into prognostic categories according to the ELN2017 criteria [6]. At the time of data collection, the current WHO 2022 and ICC guidelines had not been published. Response was assessed according to bone marrow morphology, histology, flow cytometry, and mutational analyses.

Morphological response or residual disease was assessed on day 21 by bone marrow biopsy. The threshold between residual and no residual disease was based on flow cytometry of the bone marrow collected after two cycles of induction according to the MRD criteria of the ELN [20].

### 2.2. Molecular Analyses

Peripheral blood and bone marrow samples were taken at the time of diagnosis. Mutational analysis was performed with next-generation sequencing (NGS) using the TruSight Oncology 500 Genepanel (Illumina, Berlin, Germany) to detect mutations in 1 of the 65 relevant myeloid genes, and rearrangements were assessed using the Oncomine Myeloid Research Assay (ThermoFischer, Waltham, MA, USA) using the Ion Torrent S5 panel on bone marrow aspirate or peripheral blood samples. Additional cytogenetic analysis and FISH rearrangements were performed. Cytogenetic risk groups were defined according to the European Leukemia Net (ELN) 2017 AML risk classification [6].

### 2.3. Covariates

All individual-level variables for eligible patients included demographics, treatment regimes, adverse events, and overall survival.

Treatment consisted of chemotherapy induction. In the first and second induction, this consisted of an anthracycline backbone (idarubicin, daunorubicin) combined with cytarabine; individual patients received a combination of cladribine with cytarabine. Patients receiving other therapies, such as venetoclax, azacitidine, decitabine, gilteritinib, or enasidenib, were excluded from the analysis of the induction phase but were considered for the analysis at relapse.

Treatment-related adverse events were extracted from the medical health records. The duration of hospitalization included elective admissions surrounding induction treatments, but not any admissions due to other adverse events or complications of a bone marrow transplant.

### 2.4. Statistical Analysis

The median follow-up time was estimated with the reverse Kaplan–Meier method. To compare the proportions and continuous variables between groups of patients, Fisher’s exact test and the Kruskal–Wallis test were used, respectively. There was no control for multiple testing. For OS, the median was calculated by the Kaplan–Meier method, together with the 95% confidence interval (CI). Differences in OS between groups of patients were assessed using the log-rank test. All statistical analyses were performed using the SAS software version 9.4 and R version 4.3.3.

## 3. Results

### 3.1. Study Population and Baseline Clinical Characteristics

**Figure 1:**
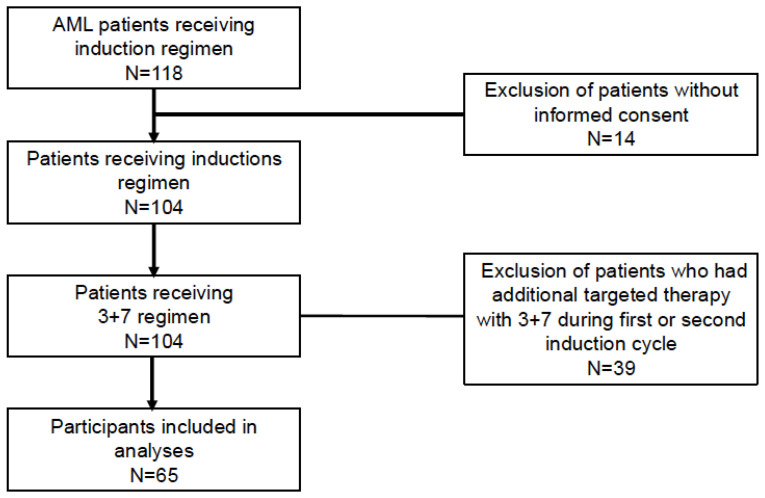
Flow diagram of patient cohort with AML.

Using data from 2017 to 2021, the complete cohort of AML patients receiving an induction regimen at the Inselspital Bern included 104 patients. Thirty-nine patients receiving additional targeted therapy were excluded. Sixty-five fully evaluated patients were included in the analysis (Figure 1). Patient characteristics at diagnosis are listed in Table 1. The median age at diagnosis was 55 (range 18–73) years old, 53 in males and 59 in females. Additionally, 62% of patients were male, and the majority were diagnosed with primary AML (73% of males, 64% of females). Almost half of the patients had an ELN 2017 adverse risk; there was no significant difference in risk between the sexes (45% males, 48% females).

Hematological values, blasts in peripheral blood and bone marrow, as well as LDH at diagnosis, did not significantly vary between males and females (Table 2); however, females had lower hemoglobin and neutrophil levels and higher thrombocyte counts than males.

### 3.2. Association Between Sex, Treatment Patterns, and Treatment Response

In the first induction, most patients received a standard 3 + 7 induction; there were no significant differences between the sexes. Furthermore, 26% of male patients also received a pretreatment with hydroxycarbamide, tretinoin, or leukapheresis, compared to only 16% of females needing these measures. For the second induction, treatment was more often idarubicin-based in females (18% vs. 5%) compared to males, who received mostly a daunorubicin-based therapy (73% bs 59%) (Table 3). Proportionally, more females went into a second induction treatment compared to males (68% vs. 55%). Among male patients, 15% required treatment for relapse at any time during the follow-up, and 23% of males died during the first or second induction chemotherapy cycles. In comparison, in the female population, 36% required treatment for relapse, and only 8% had died during induction treatments. The relapse treatment varied; however, the population size was too small to perform meaningful statistical analyses by sex.

The rate of consolidation at first remission was similar for females and males (56% vs. 60%); however, females had a lower rate of allogenic transplantations (12% vs. 25%) as consolidation therapy.

After the first induction, most patients reached morphological remission (60% male vs. 64% female), whereas more females were still alive and had not reached remission (32% vs. 25%) (Table 4). Among male patients, 15% died before their response could be assessed. Of the initial 65 patients, 38 patients were reassessed after the second induction morphologically and by using flow cytometry. The morphological response, at this point, was similar, with 91% of males and 88% of females reaching remission; however, more females had not reached morphological remission (12% vs. 5%) and were MRD positive in the flow cytometry (29% vs. 23%) (Table 4).

The median duration until regeneration of neutrophils and platelets occurred after the first cycle was around 24 days in both populations. After the second induction, hematological regeneration was significantly faster in males, with 21 days vs. 28 days for neutrophils (*p* = 0.025) and 27 vs. 37 days for thrombocytes (*p* = 0.02).

### 3.3. Association Between Sex and Toxicity

Chemotherapy-related adverse events (AE) were present in 98% of those receiving either one or two induction treatments; one patient had no documented AEs, but died within 14 days of diagnosis. The rate of AEs was similar between the sexes; however, females had significantly higher rates of neutropenic colitis (100% vs. 80%, *p* = 0.019) within the first induction cycle. The numerical sex differences in AEs were more pronounced after the second induction cycle; males had higher rates of bacteraemia (73% vs. 59%) and mucositis (18% vs. 12%) as opposed to higher rates of neutropenic colitis (54% vs. 88%), fungal infections (9% vs. 18%), and bacteriuria (5% vs. 12%) in the female population (Table 5). These differences were, however, not statistically significant. The median duration of hospitalization was similar in both cohorts, with 28 vs. 29 days for the first induction cycle and 24 vs. 26 days for the second induction cycle. For the second cycle, however, there was a significant difference in the range of duration of hospitalization between males (18–32 days) and females (22–51 days, *p* = 0.039) (Table 6).

### 3.4. Association Between Sex and Overall Survival

**Figure 2:**
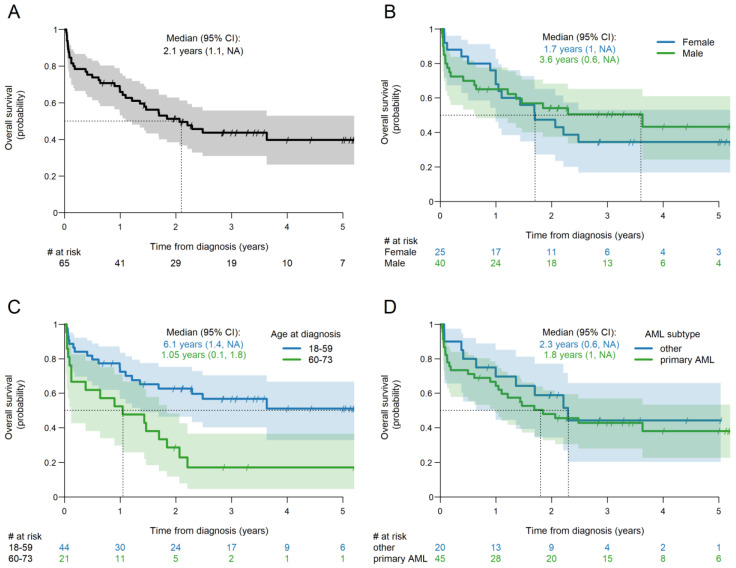
Kaplan–Meier plot of OS. (**A**) All patients; (**B**) by patient sex; (**C**) by age at diagnosis; (**D**) by AML subtype. NA = not reached. # = number.

The median overall survival was 2.1 years (95% CI 1.1-NA) (Figure 2). There were no significant OS differences by sex, age; however, this significantly impacted OS. The median OS of patients aged 18–59 was significantly longer compared to patients 60–73 years old (6.1 vs. 1.0 years, *p* = 0.003). 

## 4. Discussion

To our knowledge, this is the first real-world analysis that examined the outcome of AML patients treated with intensive 3 + 7 induction therapy, shedding light on the sex-based differences in treatment-associated toxicities. We report a significantly higher rate of neutropenic colitis among female patients. In line with a greater toxicity rate, the hospitalization duration after the second induction cycle was significantly longer for female patients in our cohort.

This is in accordance with previous analyses of clinical trials on various malignancies, including leukemia, which showed a significantly higher risk of severe adverse events, in the range of 34–50% among female patients [21]. The excessive risk in the female population is probably driven by sex differences in immune function, pharmacokinetics, and hormonal influences. For instance, chemotherapy dosing based on the body surface area, as is done for the 3 + 7 regimen, is very imprecise and does not take into account the sex differences in body composition and drug metabolism [22]. The clearance of cytrabine is significantly faster in male AML patients compared to females [23]. Interestingly, patients with higher pretreatment leucocyte or blast counts have a faster clearance because of the cytidine deaminase activity in leukemia cells [23]. In our cohort, no significant sex differences in baseline cell counts were present; the observed toxicity differences are, therefore, more likely to be due to differences in other host factors impacting drug metabolism. In a Drosophila model, exposure to cytarabin induced intestinal damage and reactive oxygen species accumulation in the gut by promoting Toll signaling and apoptotic pathways, and affected female flies more severely compared to males [24]. Thus, given the absence of a dose–response relationship for cytarabine and the interindividual pharmacokinetic variability, it is important to establish the optimal dose with the greatest therapeutic window for all patients, independent of sex [23,25].

In our cohort, OS did not differ significantly between male and female patients, but younger patients (aged 18–59) had a significantly longer OS compared to older patients (aged 60–73), confirming the results from other real-world analyses [26,27]. Also, having received two induction cycles was associated with a significant prolongation of OS compared to one induction cycle. Such findings suggest the need for tailored supportive care strategies to mitigate sex-specific toxicities and improve the tolerability of intensive chemotherapy. While these toxicities did not significantly affect OS in our cohort, they may contribute to quality-of-life differences and treatment adherence.

Emerging evidence suggests that not only the toxicity but also the efficacy of AML therapies might be sex-related. In a recent post hoc analysis of the HOVON135 trial testing 10 days of decitabine alone or in combination in older, unfit AML patients, age <76 years and female sex were associated with superior OS [28]. While the mutational profiles were comparable between younger and older patients, *ASXL1*, *STAG2*, and *U2AF1* were more prevalent in males compared to females. However, in the multivariable analysis, the cytogenetic risk, comorbidities, or physical functioning were not associated with OS [28].

Indeed, sex and age are key factors affecting the type of molecular alterations found at different disease stages. An analysis of the COSMIC database has shown that some mutations, such as *ASXL1*, *SRSF2*, and *U2AF1*, show a male predominance across all age groups, while *RUNX1* and *BCOR* are male predominant only at an older age (>60 years), and some mutations, such as *DNMT3A* and *NPM1*, are female predominant regardless of age, and some mutations, such as *PTNP11* and *WT1*, are not sex-related [29]. Given that hematopoietic stem cells and nearly all immune cells express sex hormone receptors, it is conceivable that sex hormone signaling and age-related hormonal changes contribute to differential acquisition of mutations or selection of specific clones [30,31]. Importantly, while the WHO’s fifth edition classification of hematologic malignancies [1] and the ICC [5] underscore the importance of genomic data and clinical parameters, neither explicitly addresses sex as a modifying factor. Similarly, the ELN recommendations [6,7] prioritize molecular and cytogenetic risk stratification but fail to incorporate sex as a prognostic factor.

Recent subgroup analyses of various phase 3 trials investigating targeted drugs alone or in combination with standard therapy in *IDH1* or *FLT3* mutant AML patients have shown sex-based OS differences, either favoring male or female patients [32]. However, given that clinical trials are usually not designed and do not have the statistical power to show sex differences in drug effects, it is not possible to draw conclusions for clinical practice from such post hoc analyses. In order to integrate sex and gender into clinical decision making and treatment guidelines, we need to revisit the current paradigm of drug development, dosing, and testing [33]. As a minimal requirement, clinical trial designs should ensure balanced sex representation and stratified analyses and disaggregated reporting of all trial endpoints to uncover the differential treatment responses [33]. Yet, given that drug development relies on experimental research, potential sex differences in disease biology and treatment efficacy should be considered at the level of preclinical research. A recent survey of academic researchers in cancer biology, including 26% working on hematological malignancies, found that despite the current recommendations on the inclusion of sex and gender in research design and reporting, researchers did not think that it was important to investigate sex differences in every context of cancer biology, nor in all tumor types [34]. Similarly, despite the increasing awareness of the impact of sex and gender on the clinical course of various malignancies, severe knowledge gaps exist among clinicians, as reported from a survey of Swiss hemato-oncologists [35]. Although challenging to implement currently, it will be inevitable in the future to design clinical trials that consider potential sex differences to improve the balance between toxicity and efficacy, particularly for female patients.

Our study has some limitations, including its small sample size and the exclusion of patients receiving targeted therapies. The absence of molecular and cytogenetic subgroup analyses prevents the exploration of biological mechanisms underlying the observed sex differences. Since we focused solely on the induction regimen, not enough data were collected to compare the sex differences after consolidation and hematopoietic stem cell transplant, as well as follow-up treatment in case of a relapse. Additionally, performance status, comorbidities, and infection prophylaxis, which might influence survival and treatment toxicity, were not systematically accounted for in this analysis—the lack of gender-specific data limits our understanding of sociocultural influences on AML outcomes. Gender can influence healthcare access, treatment adherence, and supportive care engagement [36]. These factors may disproportionately affect females, particularly in older age groups where caregiving responsibilities and healthcare disparities are more pronounced.

In conclusion, our analysis provides evidence of sex-based differences in chemotherapy-related toxicities among AML patients. Although these differences did not translate into significant disparities in OS, they highlight the need for further investigation into the underlying mechanisms and the potential for sex-specific risk stratification and treatment strategies. Addressing these differences could lead to more personalized and effective treatment approaches, ultimately improving outcomes for all AML patients, men and women alike.

## Figures and Tables

**Table 1:** Patient demographics and disease characteristics at diagnosis by sex. FAB classification: M0, minimal myeloid differentiation; M1, poorly differentiated myeloblasts; M2, myeloblastic with differentiation; M3, promyelocytic; M4, myelo-monoblastic; M5, monoblastic; M6, erythroblastic; M7, megakaryoblastic. Percentages may not sum to 100 due to rounding.

	Males	Females
**Number (%)**	40 (62%)	25 (38%)
**Age in years (median, range)**	53 (18–73)	59 (23–70)
**AML subtype**		
M0	2 (5%)	1 (4.0%)
M1	10 (25%)	6 (24%)
M2	7 (18%)	5 (20%)
M4	7 (18%)	4 (16%)
M5	2 (5%)	0 (0%)
M6	0 (0%)	0 (0%)
Secondary AML	7 (18%)	3 (12%)
Treatment-associated AML	2 (5%)	4 (16%)
Mixed phenotype AML	1 (3%)	0 (0%)
Undifferentiated AML	0 (0%)	0 (0%)
Extramedullary AML	2 (5%)	2 (8%)
**ELN 2017 classification**		
favorable	13 (33%)	9 (36%)
intermediate	7 (18%)	3 (12%)
adverse	18 (45%)	12 (48%)
not applicable	2 (5%)	1 (4%)

**Table 2:** Hematological values at diagnosis. * Kruskal–Wallis test.

Hematological Variables	Males (n = 40) Median (min, max)	Females (n = 25) Median (min, max)	*p*-Value *
Hemoglobin (g/L)	98 (45–170)	87 (47–140)	0.24
Thrombocytes (G/L)	58 (8–296)	79 (8–311)	0.50
Neutrophils (G/L)	1.53 (0.02–21.15)	0.94 (0.04–8.89)	0.75
Leucocytes (G/L)	5.29 (0.65–106)	6.86 (0.3–97.2)	0.84
Blasts in peripheral blood (%)	17.8 (0–84.5)	33 (0–84)	0.30
Blasts in bone marrow (%)	65 (0–95)	70 (0–95)	0.35
LDH (x ULN)	1.42 (0.56–26.2)	1.02 (0.67–10.2)	0.25

**Table 3:** Treatment patterns by sex. * Subsequent therapy may include allogeneic transplant in responding patients. Percentages may not sum to 100 due to rounding.

Treatment	Males	Females
Before first induction		
Hydroxycarbamide	8 (20%)	3 (12%)
Tretinoin	1 (3%)	1 (4%)
Leukapharesis	1 (3%)	0 (0%)
First induction 3 + 7 Regimen	40	25
Idarubicin and Cytarabine	35 (88%)	20 (80%)
Daunorubicin and Cytarabine	4 (10%)	5 (20%)
Cladribine and Cytarabine	1 (3%)	0 (0%)
Second induction 3 + 7 Regimen	22	17
Idarubicin and Cytarabine	1 (5%)	3 (18%)
Daunorubicin and Cytarabine	16 (73%)	10 (59%)
Cytarabine monotherapy	4 (18%)	4 (24%)
Cladribine and Cytarabine	1 (5%)	0 (0%)
No second induction (any reason)	18 (45%)	8 (32%)
No second induction (death)	8 (20%)	2 (8%)
Subsequent treatment for relapse *	6 (15%)	9 (36%)
Cladribine, Idarubicin, Cytarabine (Cla-Ida scheme)	0 (0%)	2 (22%)
Gilteritinib	1 (17%)	0 (0%)
Midostaurin	0 (0%)	0 (0%)
Azacitidine	0 (0%)	0 (0%)
Decitabine	3 (50%)	5 (56%)
Decitabine and Enasidenib	1 (17%)	0 (0%)
Decitabine and Venetoclax	1 (17%)	1 (11%)
Sorafenib	0 (0%)	1 (11%)
No subsequent treatment (death during the first or second induction cycle)	10 (25%)	2 (8%)
Consolidation in the first complete remission	24 (60%)	14 (56%)
Allogeneic HSCT	10 (25%)	3 (12%)
Autologous HSCT	13 (33%)	9 (36%)
Chemotherapy	1 (3%)	1 (4%)
Refusal of subsequent treatment for consolidation	0 (0%)	1 (4%)

**Table 4:** Response to treatment and hematological regeneration by sex. Percentages may not sum to 100 due to rounding.

	Males	Females	Fisher’s Exact Test *p*-Value (2-Tailed)
**Response after first induction cycle**	n = 40	n = 25	
Morphological complete remission	24 (60%)	16 (64%)	
No morphological complete remission	10 (25%)	8 (32%)	
Death before response assessment	6 (15%)	1 (4%)	
**Response after second induction cycle**	n = 22	n = 17	
Morphological complete remission	20 (91%)	15 (88%)	
No morphological complete remission	1 (5%)	2 (12%)	
MRD negative at flow cytometry	13 (59%)	10 (59%)	
MRD positive at flow cytometry	5 (23%)	5 (29%)	
No bone marrow biopsy (death before response assessment)	1 (5%)	0 (0%)	
No information on flow cytometry	3 (13.6%)	2 (11.8%)	
**Hematological regeneration after first induction cycle**	n = 40	n = 25	0.394
Neutrophils > 1 G/L reached	27 (68%)	20 (80%)	
Neutrophils > 1 G/L reached in days (median, range)	24 (18–201)	24 (16–132)	0.371
Thrombocytes > 100 G/L reached	29 (73%)	21 (84%)	
Thrombocytes > 100 G/L reached in days (median, range)	23 (17–94)	24 (18–132)	
**Hematological regeneration after second induction cycle**	n = 22	n = 17	1
Neutrophils > 1 G/L reached	20 (91%)	16 (94%)	
Neutrophils > 1 G/L reached in days (median, range)	21 (15–122)	28 (12–68)	0.64
Thrombocytes > 100 G/L reached	19 (86%)	14 (82%)	
Thrombocytes > 100 G/L reached in days (median, range)	27 (18–122)	37 (19–136)	

**Table 5:** Toxicity. Percentages may not sum to 100 due to rounding.

	Males	Females	Fisher’s Exact Test *p*-Value (2-Tailed)
**Toxicity after first induction cycle (regimen 7 + 3)**	n = 40	n = 25	
Neutropenic colitis	32 (80%)	25 (100%)	0.019
Bacteremia	36 (90%)	20 (80%)	0.288
Mucositis	6 (15%)	2 (8%)	0.471
Fungal infections	4 (10%)	6 (24%)	0.165
Bacteriuria	3 (8%)	5 (20%)	0.243
Clostridium difficile-associated colitis	3 (8%)	0 (0%)	0.279
Urosepsis	0 (0%)	1 (4%)	0.385
**Toxicity after second induction cycle (regimen 7 + 3)**	n = 22	n = 17	
Neutropenic colitis	12 (54%)	15 (88%)	0.073
Bacteremia	16 (73%)	10 (59%)	0.279
Mucositis	4 (18%)	2 (12%)	0.667
Fungal infections	2 (9%)	3 (18%)	0.644
Bacteriuria	1 (5%)	2 (12%)	0.584
Clostridium difficile-associated colitis	4 (18%)	3 (18%)	1
Urosepsis	0 (0%)	1 (6%)	0.459

**Table 6:** Duration of hospitalization. * Kruskal–Wallis test.

Duration of Hospitalization in Days	Males (Median, Range)	Females (Median, Range)	*p*-Value *
First induction 3 + 7 regimen	28 (11–68)	29 (23–61)	0.32
Second induction 3 + 7 regimen	24 (18–32)	26 (22–51)	0.039

## Data Availability

All data generated or analyzed during this study are included in this article. Further enquiries can be directed to the corresponding author.

## Abbreviations

The following abbreviations are used in this manuscript:

AML	Acute Myeloid Leukemia
MDS	Myelodysplastic Neoplasm
FAB	French American British Classification
WHO	World Health Organization
ICC	International Consensus Classification
ELN	European Leukemia Net
ASCT	Autologous Stem Cell Transplant
alloHSCT	Allogeneic Hematopoietic Stem Cell Transplant
OS	Overall Survival
APL	Acute Promyelocytic Leukemia
MRD	Minimal Residual Disease
NGS	Next Generation Sequencing
AE	Adverse Events
CI	Confidence Interval
LDH	Lactate Dehydrogenase
ULN	Upper Limit of Normal
